# Supplementary material for: SyNDI: synchronous network data integration framework
Source: BMC Bioinformatics. 2018 Nov 6;19:403. doi: 10.1186/s12859-018-2426-5 (PMC6219086; doi:10.1186/s12859-018-2426-5)
Supplement: Supplementary file 5 — Files of the example presented in Additional file 4. This additional file is a zip package that contains all files used in Additional file 4. Detailed descriptions of each of these files are presented in a README file included in this zip package. (GZ 30411 kb) [file 12859_2018_2426_MOESM5_ESM.gz › SyNDITools/Meme2Fimo/meme_4.9.0_4.tar.gz!/meme_4.9.0/website/html/glam2-intro.html]

GLAM2 - Overview


|  |  |
| --- | --- |
|  | MEME Suite The MEME Suite web application requires the use of JavaScript   Javascript doesn't seem to be available on your browser.  Overview  GLAM2 is a tool for discovering *gapped* motifs in a group of DNA or protein sequences.  Equivalently, it is a tool for aligning similar regions in a group of sequences.  It is harder to discover gapped motifs than gapless motifs: so when you use GLAM2, we recommend that you also do a simpler gapless analysis with MEME.  Here is a GLAM2 sample output. A motif consists of aligned columns (coloured letters) and insertions (grey letters). The aligned columns may include deletions (black dots). GLAM2 does not try to align inserted (grey) letters with one another: it assumes their identity is unimportant.  GLAM2 reports a score for each motif, higher scores indicating stronger motifs. It also reports a "marginal score" for each site, stronger scores indicating better matches to the motif.  Unlike MEME, GLAM2 does not try to find several different motifs all in one go. Instead, it performs replicates: it tries to find the best possible motif ten times. The highest-scoring result is shown at the top, with the other replicates below it. You should check that at least some of the replicates are similar to the top motif: if not, click the button to re-run GLAM2 more slowly and thoroughly.  You can control the gapiness of GLAM2 motifs, using four "pseudocount" options. Their relative values control GLAM2's aversion to gaps: increasing the no-deletion pseudocount relative to the deletion pseudocount makes it more averse to deletions, and likewise for the no-insertion and insertion pseudocounts. Their absolute values control GLAM2's preference for putting gaps together in the same positions: decreasing the deletion and no-deletion pseudocounts makes it more prone to gather deletions into a few columns, and likewise for the (no-)insertion pseudocounts. The pseudocounts affect the score calculation, so scores are not comparable between motifs found with different pseudocounts.  If your sequences include repeats or extended regions of homology, GLAM2 will gladly find them for you.  GLAM2 will always report the best motif it can find, even if you give it random sequences. So, to check that your motif is significant, you may wish to run GLAM2 on control (e.g. shuffled) sequences as well, and compare the motif scores. The input form includes a checkbox for shuffling the sequences. |
